# Development, validation and assessment of the test on knowledge about basic life support and use of automated external defibrillator among schoolchildren

**DOI:** 10.1186/s13049-019-0683-6

**Published:** 2019-12-23

**Authors:** Vesna Borovnik Lesjak, Andrej Šorgo, Matej Strnad

**Affiliations:** 1 Center for Emergency Medicine, Health Center Maribor, C. Proletarskih brigad 21, 2000 Maribor, Slovenia; 2 Faculty of Natural Sciences and Mathematics, University of Maribor, Koroška cesta 160, 2000 Maribor, Slovenia; 3 Faculty of Electrical Engineering and Computer Science, University of Maribor, Koroška cesta 46, 2000 Maribor, Slovenia; 4 Faculty of Medicine, University of Maribor, Taborska ulica 8, 2000 Maribor, Slovenia

**Keywords:** Automated external defibrillator, Cardiopulmonary resuscitation, Knowledge, out-of-hospital cardiac arrest, Schoolchildren, Validation study

## Abstract

**Background:**

Educating lay public can significantly strengthen the Chain of Survival after out of hospital cardiac arrest. Schoolchildren are an accessible population for learning basic life support (BLS) and use of an automated external defibrillator (AED) and can be regarded as multipliers of knowledge that can reach the whole population. This study aimed to develop and validate a test for examining levels of knowledge about BLS and AED among schoolchildren that can be used to uniformly present reliable data.

**Methods:**

A knowledge test about BLS and AED consisting of 10 multiple-choice questions was developed and implemented before and after a 2-h BLS and AED course consisting of an interactive lecture and a practical workshop for 783 students in seventh and ninth grades of elementary schools in Maribor, Slovenia. Each question was analyzed and presented with descriptive statistics and educometric parameters (difficulty and discriminating indices). All variables were checked for normality with the Kolmogorov-Smirnov test and analyzed using non-parametric tests. Statistical significance of the differences in knowledge before and after intervention were calculated with chi-square statistics and effect sizes *r* are reported. Differences between genders, grades and previous attendance to BLS courses were compared using Mann – Whitney *U* test. The effect size was calculated from the *Z* score and reported as *r* value.

**Results:**

After educometric analysis, questions were adjusted to meet the requirements of satisfactory functioning difficulty and discriminating indices (values between 0,40 and 0,60, and above 0,20, respectively). Only one question had to be eliminated due to inadequate difficulty and discriminating index (0,99 and 0,02, respectively). Measurement invariance across gender (*p* < 0,001), school grade (*p* < 0,001), and attendance to previous courses (*p* = 0,303) was assured.

**Conclusions:**

A test for accurate and reliable measurement of knowledge of BLS and AED among schoolchildren was developed and validated. According to the findings it can now reliably be used to assess baseline knowledge and potential improvement in knowledge after a course on BLS and AED. Standardized data gathered with a validated tool can now be presented at legislative levels to promote BLS and AED courses implementation in school curricula.

## Background

Survival after out-of-hospital-cardiac-arrest (OHCA) remains poor, around 10% [1, 2] despite the very high-level professional care for these patients [3]. The first three links of the Chain of Survival need to be strengthened by raising awareness about cardiac arrest in the community and demonstrating to the lay public the importance of early recognition, cardiopulmonary resuscitation (CPR), and use of an automated external defibrillator (AED).

Systematic basic life support (BLS) training in schools has been shown to be an effective stimulation for laypersons’ action in cases of OHCA [4]. Training schoolchildren is easy and cost-effective, especially if trainings are part of compulsory school activities, and is therefore encouraged worldwide [5, 6]. In 2015, the World Health Organization endorsed the international interdisciplinary “Kids Save Lives” initiative that promotes CPR training and courses in schools for children of 12 years and older [7]. As education in Europe is not centralized such initiatives and solutions depend on the motivation of individual countries. For example, in five European Union countries (Belgium, Denmark, France, Italy, and Portugal) CPR education is mandated by law, in 16 countries (including Slovenia), CPR education is merely a suggestion [8]. In Slovenia, there are numerous initiatives for transferring this important knowledge to elementary school children. Individual regions and schools in Slovenia do partake in voluntary BLS courses offered by the National Institute for Public Health or other regional organizations. Through such agreement such courses become part of the compulsory out of the school-curricula, so providers of the course do not need to deal with formalities such as written consents of the parents, incorporation in school schedules etc.

CPR training in schools is a vital part of disseminating knowledge and positive attitude towards CPR among laypersons [9]. It has long since been established that schoolchildren are particularly susceptible and motivated for learning CPR and can be easily and quickly taught [10–12]. Also, the sooner the continuous age-appropriate tuition is introduced the more sustainable the knowledge will be [13]. The importance of educating a wide population base [14] and of imparting a positive attitude towards CPR [15] has also been recognized for some time now. Children serve as potential multipliers of CPR knowledge and positive attitude towards action in cases of OHCA in their environment [16]. CPR training also potentially leads to perceiving CPR knowledge as a wanted skill later in life [9].

The rising importance of bystander CPR and emphasis on schoolchildren recruitment has led to increasing availability of courses for children of school ages. Much attention is paid to knowledge gain after such courses but a structured and validated approach is missing [17–19]. Thus, a need for a comprehensive and educometrically sound standardized measurement instrument to assess BLS and AED knowledge among schoolchildren remains. Existence of such tests will additionally allow comparative studies of effectiveness of different BLS and AED courses.

### Aims and objectives

The ultimate aim of the authors is development of a course for elementary school students to raise awareness, knowledge and skills for application of BLS and AED in real life situations. To be able to assess outcomes of such a course, the first task was development of validated and reliable instrument to measure knowledge on BLS and AED described in this paper.

Our study was divided into two parts as follows. The objective of the exploratory Study 1 was to develop an appropriate instrument to accurately measure knowledge about BLS and AED (Objective 1).

After exploration of the test characteristics the objective of the confirmatory Study 2 was to test the instrument across gender, two different grades, and attendance to previous BLS and AED courses to ensure measurement invariance (Objective 2).

## Methods

### Description of the course

The course was organized by the Center for Emergency Medicine of the Maribor Health Center in cooperation with the City of Maribor Municipality, which financed the courses for all of 20 interested public elementary schools in the Municipality of Maribor.

The course was designed for individual classes consisting of up to 30 seventh- and ninth-grade students and was identical throughout the study. The course was divided into 2 parts, each lasting one academic hour (45 min). In the first part, an emergency physician gave students an interactive lecture on principles of BLS and AED. The lecture was supported by an overhead presentation. The course was continued with a practical workshop where each student practised on their own training torso manikin (Prestan Professional Adult Manikin, Prestan, Mayfield Village, OH, USA) along with an AED prop – a cardboard sample with adhesive paper electrodes. A single real training AED (Defibtech Trainer AED, Defibtech, Guilford, CT, USA) was used for guidance and cardiac arrest simulation.

### Sample

Sampling was based on the incidental application of elementary schools in the time frame of the studies, that is from November to December 2017 and from October to December 2018 for Study 1 and Study 2, respectively.

The sample in Study 1 consisted of 172 students (116 seventh- and 56 ninth graders) from three elementary schools in the city of Maribor, who fulfilled both the pre- and post-course questionnaire. Of those, 82 were boys, and 90 were girls.

Six hundred and eleven students from 10 schools were included in Study 2. Of those, 283 were seventh- and 328 ninth graders. There were 310 boys nad 299 girls, two students refrained from stating their gender. Only a minority of students has received any prior BLS training (31 and 148 students from Study 1 and 2, respectively).

The average age of children in seventh and ninth grade of the 9-years compulsory elementary schools in Slovenia is 12 and 14 years, respectively.

### Measuring instrument

The instrument consisted of two parts.

- The first part contained demographic data (gender, school grade, attendance to previous BLS courses).
- The second part was a knowledge test consisting of ten multiple-choice questions addressing theoretical knowledge about BLS and AED. The wording has been carefully selected to conform to this population’s level of understanding and interpretation. Each question offered 5 answers of which only one was correct, therefore allowing students to achieve a maximal score of ten points. The fifth answer in all cases was “I do not know.” The answer was included to prevent guessing, however, it was treated as incorrect in later analyses.

Questionnaires were handed out immediately before the lecture and immediately after the practical workshop. The content of the paper and pencil test was identical before and after the course. In Study 2, the content of the test was modified according to the analyses of Study 1 results. The test took students about 5 min to fulfil in both studies. The questionnaire did not require the provision of a name to assure anonymity. However, students were asked to come up with individual codes that would be used on the pre- and post- course test for the purpose of pairing tests of the same individuals for further analyses. Each student had to state his or her agreement with fulfilling the questionnaire (see Additional file 1). A request for approval of the National Medical Ethics Commitee had been filed and granted.

### Statistical analyses

Full text of the applied knowledge test is provided in the Additional file 1. Complete list of frequencies of the answers along with mode, mean, and standard deviation is provided in the Additional file 1: Table S1 section. In the text (Tables 1 and 2) sums of correct answers gained in pre- and post-test, differences and educometric indices are reported.

**Table 1:** Analysis of the knowledge test in Study 1 (*N* = 172)

Question	Sum correct - Pre	Sum correct - Post	Δ_Pre-Post_	χ^2^	*p*	r	Correct - Pre (%)	I_diff_	I_discr_
6. How is basic life support correctly performed?	53	172	119	170,86	< 0,001	0,71	30,3	0,32	0,43
10. What is an AED (automatic external defibrillator)?	70	152	82	85,56	< 0,001	0,51	40	0,43	0,72
9. What do you do if you are unsure whether a person is in cardiac arrest or not?	71	128	57	36,74	< 0,001	0,33	40,6	0,43	0,32
8. How do you perform artificial breaths in an unconscious person?	110	154	44	33,50	< 0,001	0,31	62,9	0,65	0,55
3. A person suddenly loses consciousness and collapses. What do you do?	101	145	44	26,63	< 0,001	0,28	57,7	0,58	0,66
7. On the sketch of the torso below mark with a cross the correct site for chest compressions during basic life support;	72	115	43	21,16	< 0,001	0,25	41,1	0,43	0,43
2. Who can help in a case of cardiac arrest?	143	168	25	20,79	< 0,001	0,24	81,7	0,82	0,31
4. How do you check if a person is breathing normally?	145	164	19	9,41	0,002	0,17	82,9	0,84	0,31
1. How do you recognize a person in cardiac arrest?	113	132	19	4,06	0,044	0,11	64,6	0,67	0,34
5. What kind of breathing is NOT a sign of life?	172	173	1	2,00	0,157	0,08	98,3	0,99	0,02

Note: df = 1. Pre – pre-test; Post – post-test; Δ_Pre-Post_ - differences between pre- and post-test; χ^2^ – chi square; r - effect size; I_diff_ - difficulty index; I_discr_ – discriminating index

**Table 2:** Analysis of the knowledge test in Study 2 (*N* = 611)

Question	Sum correct - Pre	Sum correct - Post	Δ_Pre-Post_	χ^2^	*p*	r	Correct - Pre (%)	I_diff_	I_discr_
6. How is basic life support correctly performed?	318	585	267	308,17	< 0,001	0,50	52,4	0,52	0,67
10. What is an AED (automatic external defibrillator)?	272	521	249	239,63	< 0,001	0,45	45	0,45	0,78
9. What do you do if you are unsure whether a person is in cardiac arrest or not?	196	398	202	146,05	< 0,001	0,35	32,4	0,32	0,30
3. A person suddenly loses consciousness and collapses. What do you do?	390	559	169	129,47	< 0,001	0,33	64,7	0,65	0,57
8. How do you perform artificial breaths in an unconscious person?	397	561	164	124, 60	< 0,001	0,32	66,1	0,66	0,60
7. On the sketch of the torso below mark with a cross the correct site for chest compressions during basic life support;	292	425	133	55,64	< 0,001	0,22	49,7	0,50	0,33
5. What kind of breathing is NOT a sign of life?	391	514	123	68,93	< 0,001	0,24	64,5	0,65	0,63
2. Who can help in a case of cardiac arrest?	490	575	85	54,01	< 0,001	0,21	80,5	0,80	0,49
4. How do you check if a person is breathing normally?	517	566	49	20,00	< 0,001	0,13	84,9	0,85	0,39
1. How do you recognize a person in cardiac arrest?	416	451	35	4,33	0,037	0,06	68,5	0,69	0,35

Note: Questions are sorted by increasing difficulty. Df = 1. Pre – pre-test; Post – post-test; Δ_Pre-Post_ - differences between pre- and post-test; χ^2^ – chi square; r - effect size; I_diff_ - difficulty index; I_discr_ – discriminating index

All variables were checked for normality with the Kolmogorov-Smirnov test (*p* < 0,05). Because assumption of normality was not met non-parametric statistics was a choice. Statistical significances of the differences in knowledge before and after intervention were calculated with chi-square statistics and effect sizes *r* are reported. Differences between groups (boys and girls, different grades, previous attendance to BLS courses) were compared using Mann – Whitney *U* test. A 2-sided *p* < 0,05 was considered significant. The effect size was calculated from the *Z* score and reported as *r* value. Margins were set according to Cohen at small (0,1), medium (0,3), and large (0,5) [20].

The item’s difficulty index was calculated as a percentage of the total number of correct responses to the test item [21] and ranges from 0 to 1 [22]: less than 0,20 being too difficult; 0,40 to 0,60 excellent; and more than 0,90 being too easy; Discriminating indices were used to determine the sensitivity of items and test as a whole to measure unitary ability. The sample was divided into thirds. The index was then calculated by subtracting appropriate test item difficulty indices of the lower third from the difficulty index of the test item of the upper third [22]. The higher the coefficient, the more discriminative the item: if the value of the discriminative index is ≥0,40, then the item is functioning satisfactorily; if it is between 0,30 and 0,39, then little or no revision is required; if between 0,20 and 0,29, then the item is marginal and needs revision; and if it is equal or below 0,19, then the item should be eliminated or completely revised [21].

SPSS software (SPSS Inc., Chicago, IL, USA) was used for statistical analysis. Effect sizes were calculated by using online tools [23, 24].

### Limitations of the study

Courses were lead by 3 different instructors and their assistants (registered nurses working as paramedics) for the theoretical and practical parts, respectively. The content of the course was predetermined and based strictly on the latest resuscitation guidelines with which the instructors, all of which are emergency physicians - and their paramedic assistants are perfectly familiar with as per their profession. Therefore, there could not be any inter-instructor variability in this respect. Even though the authors have acquired all reasonable measures to alleviate possible bias, the sole process of educating and training includes a variety of uncontrollable factors, such as personal attractiveness, assertiveness, communication skills of the instructor on one hand, and students’ momentary and general attentiveness on the other. However, the sum of all factors’ variability reflects the real-life situation where a number of instructors will be involved in the process.

## Results

### Study 1 – exploratory phase

The results of the test are presented in Table 1 and are sorted by increasing the difficulty of the questions (in Additional file 1).

Results of the educometric qualitative analysis of the knowledge part of the pre-test items itself showed that four (no. 3 - *»A person suddenly loses consciousness and collapses. What do you do?«*; 7 - *»On the sketch of the torso below mark with a cross the correct site for chest compressions during basic life support:«*; 9 – »*How do you perform artificial breaths in an unconscious person?«*; and 10 – »*What is an AED (automatic external defibrillator)?«*) out of 10 questions had an excellent difficulty index, only one (no. 5 – *»Who do you call if you witness a cardiac arrest?«*) was too easy. That same question also had a very low discriminating index on the pre-test, confirming its abundance. According to discriminating indices, half of the questions functioned satisfactorily and 4 need little or no revision on pre-test (Table 1).

### Study 2 – confirmatory phase

Based on the results of Study 1 only one adjustment was made. Question no. Five was replaced with another relevant question about agonal breathing (“*What kind of breathing is NOT a sign of life?”*)*.*

Due to the larger sample size (*N* = 611) the described method for calculating discriminating indices had to be revised because the distribution of a sample into thirds based on overall achieved score was unequal. Therefore the sample was divided into fifths, using the top 20% of students (*n* = 134) and bottom 20% (*n* = 163) for calculations of discriminating index. All questions had excellent difficulty indices except no. 9, which had an as good as acceptable index (Table 2). The ability to discriminate between top and bottom scoring students were satisfactory for 6 out of 10 questions (Table 2). No item needed revision incumbently or had to be eliminated.

Following educometric analysis of each question, analysis of the test as a whole was performed to ensure measurement invariance across gender, school grade and previous attendance to courses on BLS and AED. Differences in sums of correct answers on pre- and post-test were statistically significant between boys and girls, and seventh and ninth grades. However, previous attendance to any kind of BLS and AED course had no effect on the test score (Table 3). Of 603 students that stated their gender and fulfilled both pre- and postcourse test 309 were boys and 294 were girls. Of 608 students that fulfilled both the pre- and post-course test 280 were 7th – and 328 were 9th graders. Of the 85 students that provided an answer, 62 students attended some kind of a BLS and AED course in the past and 23 received no prior training in BLS and AED.

**Table 3:** Measurement invariance of the knowledge test in Study 2

	Correct answers	Mann-Whitney U	Z	*p*	r
Gender	Pre-test	36,561,5	- 4,19	< 0,001	0,17
	Post-test	36,522,5	- 4301	< 0,001	0,18
School Grade	Pre-test	33,740	− 5704	< 0,001	0,23
	Post-test	35,103	− 5178	< 0,001	0,21
Attendance to previous courses	Pre-test	610,5	− 1031	0,303	0,11
	Post-test	617,5	−0,995	0,32	0,11

Note: Z – z value; r - effect size

## Discussion

The aim of this study was to develop and validate an instrument to reliably measure knowledge of BLS and AED among schoolchildren. With these instruments, educators will be able to indirectly measure relevance and effectiveness of their BLS and AED courses and subsequently modify their courses accordingly, if necessary. The topic of CPR education has namely become a centre of attention, included also in the latest resuscitation guidelines, as resources for postresuscitation care of OHCA victims become exhausted. Education of schoolchildren has become a major topic in promoting lay public participation in enhancing survival after OHCA. Implementing BLS and AED courses in school curricula would be most efficient, but argumentation presented to national governments and offices need to be supported by reliable and validated data in order to gain their support. Despite extensive data on capabilities and willingness of schoolchildren for learning and doing BLS and AED most countries still lack national policies on the matter [8].

Thus far, published studies have used a variety of questionnaires and surveys on BLS and AED knowledge and thereby indirectly measured course’s effectiveness among schoolchildren [17–19, 25], but there remained a lack of a uniform, validated instrument to accurately measure actual knowledge (baseline and after a course) about BLS and AED. Such an instrument needs to comply with the specifics of this population. Understanding and interpretation of specific phrases and words concerning cardiac arrest, BLS and AED in this population was taken into account when developing this questionnaire. Nevertheless, even among children of the same age, different levels of understanding are to be expected based on variable scholastic achievements and pre-course knowledge about the topic. The meaning of words (e.g. unconscious, bystander etc.) is namely constructed by the actual context in which children hear these words. By multiplying the communication experience with these words a mature concept is formed leading to the same meaning to different people [26]. Therefore, an inherent bias with different understanding of specific words exists among children and between children and adults because exposure to such wording has so far been variable. The post-course gain in knowledge can also be interpreted as formation of previously unknown concepts of specific wording in regard to cardiac arrest and BLS and AED which also ensures meaningful transfer of reliable information both ways during an emergency call with a dispatcher.

Our knowledge test in Study 1 was tested on a pilot sample [27] (*N* = 172) and reflected a need for certain changes. After modification, it was tested again on a larger sample (*N* = 611) in Study 2 and the educometric analysis was satisfactory and the test in the latest form was validated for use among schoolchildren.

With regard to Objective 2, this study revealed that girls scored better on the test than boys which was to be expected according to previous meta-analysis findings on gender differences in scholastic achievements [28]. Not surprisingly, ninth-graders did better on the test compared to seventh graders. Effect sizes were small, possibly indicating that differences were generic, namely due to inherent differences between genders’ scholastic achievements and age rather than an indicator of the test’s measurement discrimation. Measurement invariance of the developed instrument across gender and two different grades can therefore intuitively be applied. Whether or not students had beforehand attended a course on BLS and AED had no effect on the test score, indicating that the present knowledge test is applicable for all students regardless of baseline knowledge. These findings could also implicate that previously attended courses had no measurable medium- to long-term effect as long-term studies are lacking [13], which is one of the main research concerns in the wider scope of the research work of the authors and needs to be explored in the future.

## Conclusions

In regard to Objective 1, this study provided a knowledge test with carefully selected questions on BLS and AED covering all important topics that are useful in bolstering the first three links in the Chain of Survival. Quality analysis of the questions satisfied all educometric requirements. In regard to Objective 2, the developed measuring instrument for assessing knowledge functioned as expected, namely following known differences among boys and girls and different grades, and showing indifference regarding attendance to previous BLS and AED courses.

This research enriches previous studies on BLS and AED knowledge among school children by providing a comprehensive and educometrically sound standardized measurement instrument to assess BLS and AED knowledge among schoolchildren. The instrument shows compelling educometric properties, validity, and measurement invariance across two grades, genders, and attendance to previous courses and can now be implemented for future BLS and AED course analyses. And lastly, it ensures that proposals to implement these courses in nationwide school curricula are credible.

## Supplementary information

**Additional file 1.** The Questionnaire and Table S1 (Complete frequency analyses of the pre- and post-test in Studies 1 and 2).

## Data Availability

The datasets used and/or analysed during the current study are available from the corresponding author on reasonable request.

## Abbreviations

AED: Automated external defibrillator
BLS: Basic life support
CPR: Cardiopulmonary resuscitation
EMS: Emergency medical services
OHCA: Out-of-hospital-cardiac-arrest
